# The prevalence of thoracic aorta aneurysm as an important cardiovascular disease in the general population

**DOI:** 10.1186/s13019-022-01767-0

**Published:** 2022-03-23

**Authors:** Entezar Mehrabi Nasab, Seyyed Shamsadin Athari

**Affiliations:** 1 Department of Cardiology, School of Medicine, Tehran Heart Center, Tehran University of Medical Sciences, Tehran, Iran; 2 Department of Immunology, School of Medicine, Zanjan University of Medical Sciences, Zanjan, Iran

**Keywords:** Aneurysm, Dissection, Heart disease, Echocardiography

## Abstract

**Background:**

The aorta is the largest and main artery in the body. The enlargement of the aortic diameter known as ectasia results in aneurysm. Thoracic aorta aneurysm can involve one or more segments of the aorta. Non-invasive imaging techniques play an important role in identifying patients, estimating maximal aneurysm diameter, following up patients, and detecting complications. So, this study was performed to estimate the prevalence of ascending thoracic aorta aneurysm in the general population of Iran.

**Methods:**

People with an abnormal aortic size (˃ 36 mm) were enrolled and subjected to diagnostic tests, and related risk factors were assessed.

**Result:**

Of the 3400 people examined, 410 (12%) had abnormal aorta sizes, and 42 (1.2%) had ascending aorta aneurysm. Out of the 410 patients with elevated aorta size, 235 (57%) were males, and 175 (43%) were females. Overall, 229 patients (56%) had hypertension, and 255 (62%) were over 60 years old.

**Conclusion:**

In this study, we showed that the prevalence of ascending aorta aneurysm in the general population of Iran was about 1.2%. Ascending aorta aneurysm is a threatening pathology of the aorta. The high prevalence of hypertension may explain the high incidence of aneurysm in our studied population. Therefore, it is necessary to implement an accurate screening plan to identify patients with hypertension and provide appropriate treatment and adequate follow up to patients. Patients with ascending aorta aneurysm are also recommended to modify their lifestyles.

## Introduction

The aorta is the largest and main artery in the body and consists of two arteries; the thoracic and the abdominal. The thoracic aorta is divided into three regions including ascending aorta, aortic arch, and descending aorta. The aortic root includes the aortic valve, as well as annulus and sinus of Valsalva. The ascending aorta extends from the Sino tubular junction to the first arch vessel. The aortic arch comprises of the innominate, left common carotid, and left subclavian artery, as well as the ligamentum arteriosum at the end of the arch. The descending aorta begins at the end of ligamentum arteriosum and continues to the diaphragm. The aorta size increases during life [1, 2], and the normal size of aorta depends on age, sex, and body surface [3].

Ectasia refers to the enlargement of the aortic diameter to at least 50% of the normal range, which results in aneurysm formation when the ectasia exceeds tolerance limits [4]. TAA can involve one or more segments of the aorta. Overall, 60% of TAA cases involve the aortic root and the ascending aorta, 40% inflict the descending aorta, and 10% are identified in each of arch or the thoracoabdominal segment [5]. TAA is divided into three categories based on the underlying cause; degenerative, inflammatory, and hereditary. However, TAA may be detected in the context of a variety of other disorders [6]. There are several risk factors for the growth and rupture of ATAA. These include a familial history of the disease, aortic dissection, advanced age, hypertension, COPD, cigarette smoking, male gender, and elevated aortic diameter [7]. ATAA is a subtle, indolent, and dangerous disease [8], and most patients are asymptomatic at the time of diagnosis [5].

Non-invasive imaging techniques play an important role in identifying TAA patients, estimating maximal aneurysm diameter, following up patients, and detecting complications. TTE is used to monitor TAA and can provide a clear image of the aortic root, ascending aorta, the arch up to isthmus, and some portions of the descending and proximal abdominal aorta. TEE can be used for conducting a complete evaluation of aortic function [9, 10]. CTA and MRA are also among common imaging techniques that are used for evaluating TAA, which obviates the limitations of acoustic methods [11].

The incidence of TAA is 5–10 per 100,000 person-years [12]. Approximately, 15,000 people in the United States and 30,000 in Europe are diagnosed with ATAA each year [13]. Limited studies have been performed on the prevalence of ATAA, and determining the prevalence of this disease is essential for delivering quality and cost-effective care. According to our literature search, there are no large-scale comprehensive studies to estimate the prevalence of ATAA in the general population of Iran. So, this study was performed to estimate the prevalence of ATAA in an Iranian population.

## Methods

In this study, the patients were initially evaluated by TTE, and then CTA was performed in patients with an ascending aortic size of greater than 45 mm.

Overall, 3400 people from the west region of Iran were randomly selected and subjected to TTE. The study had a double-blinded design, and all TTEs were performed by the same cardiologist. The study was conducted over a period of one year from August 2019 to August 2020. The patients’ ages ranged between 18 and 90 years. The normal size of the ascending aorta was defined as 2.7 ± 0.4 for females and 3 ± 0.4 for males. An ascending aortic size of ˃ 36 mm was considered as abnormal. Aortic aneurysm is technically defined as vessel dilation greater than 50% above the normal diameter of the aorta [14]. In this study, we considered an aorta size larger than 45 mm as aneurysm.

## Result

The participants were examined at two stages. First, aortic size was determined, and those with an abnormal aortic size (i.e. ˃ 36 mm) underwent additional diagnostic tests. Then patients with aortic aneurysm (˃ 45) underwent CTA and echo imaging to confirm aortic size. All the participants were further assessed for identifying potential risk factors.

**Fig. 1:**
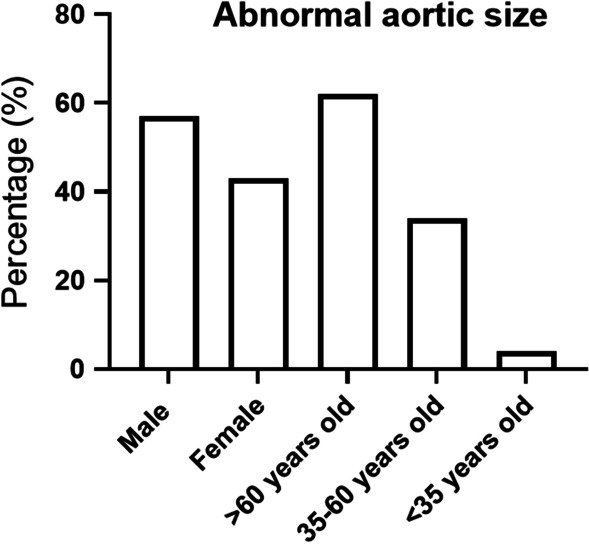
The 410 persons had abnormal aorta size who were separated and showed according to demographic factors

Among 3400 people evaluated, 410 (12%) had abnormal aorta sizes (out of normal range) and 42 (1.2%) were identified with ATAA. Out of 410 people with abnormal aortic size, 235 (57%) were males, and 175 (43%) were females. In addition, 255 (62%), 139 (34%), and 16 (4%) were over 60, 35 to 60, and under 35 years old, respectively. Hypertension was detected in 229 (56%), and 73 (18%) of the patients had coronary artery disease. Histories of open coronary artery surgery and coronary artery stent were found in 6% and 4.4%, respectively (Fig. 1).

**Fig. 2:**
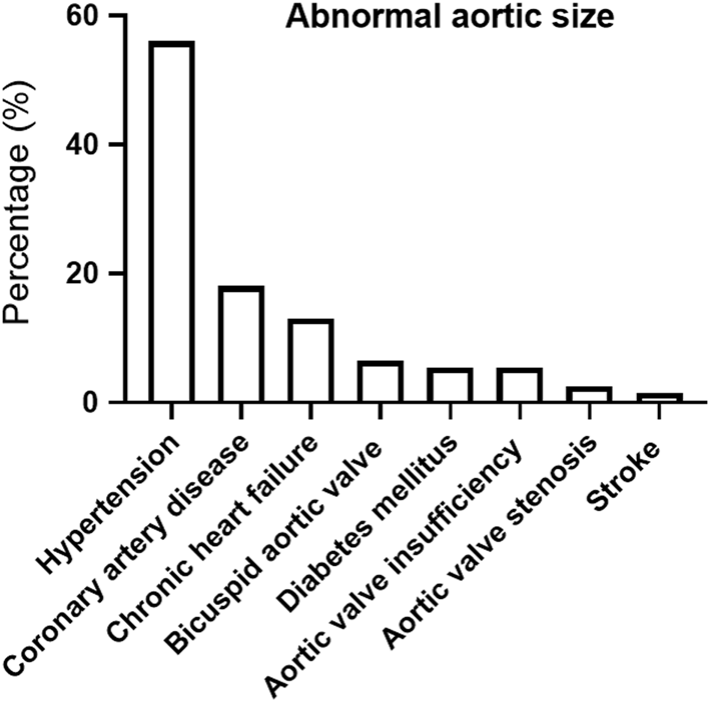
The 410 persons had abnormal aorta size who were separated and showed according to related risk factors and diseases

Fifty-four (13%) patients had chronic heart failure, and 6 (1.5%) had a history of stroke. This is while 22 (5.4%) patients had diabetes mellitus. Overall, 26 (6.3%) patients were diagnosed with bicuspid aortic valve (BAV) of whom 16 had lower than 35 years old. Aortic valve insufficiency was identified in 23 (5.6%) patients, and 11 (2.6%) had aortic valve stenosis (Fig. 2).

**Fig. 3:**
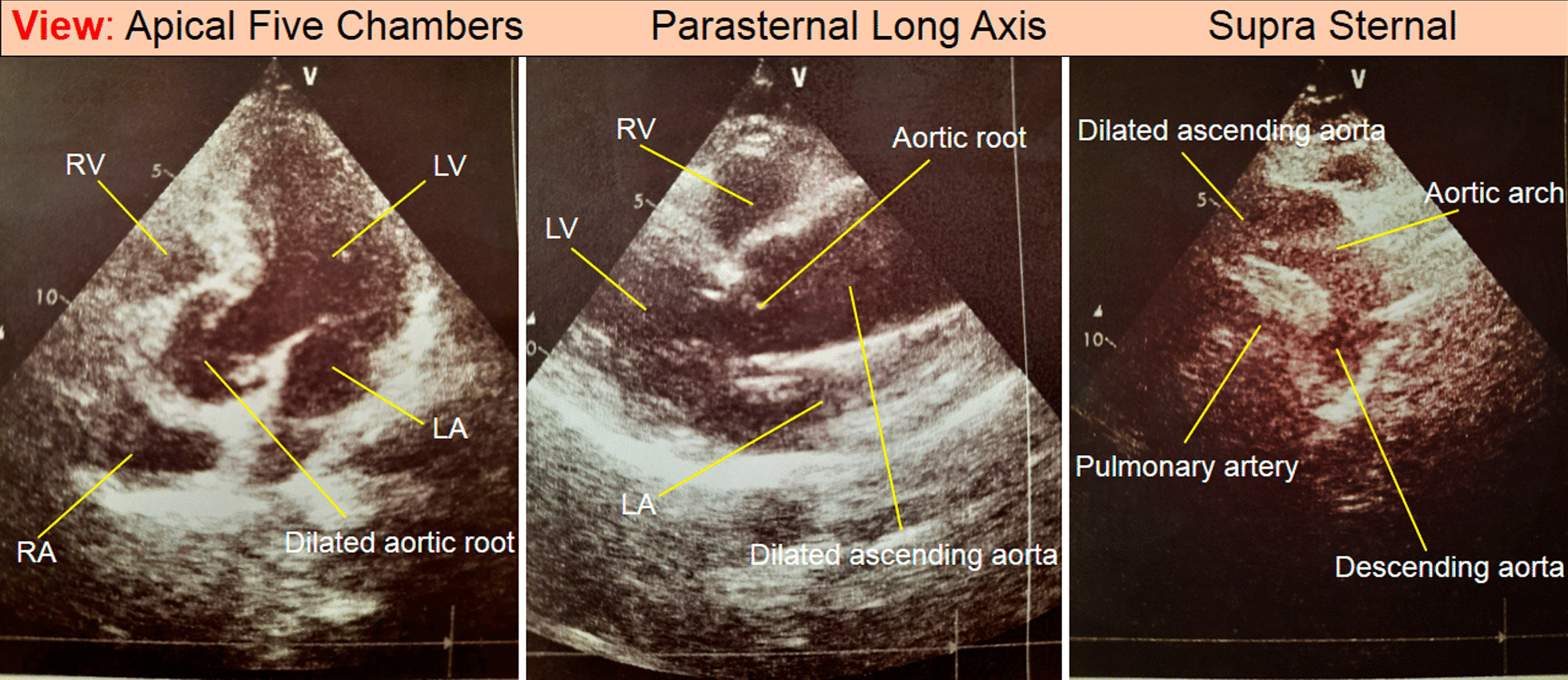
Echocardiography showed aortic aneurysms and related abnormalities

Smokers constituted 22 (5.4%) of the participants. One patient had tetralogy of Fallot, one had Marfan, and two had aortic dissection. A positive family history of ATAA was reported in 5 of the patients. Finally, among 42 patients with ATAA, 25 (60%) were over 60 years old, and 23 (55%) had hypertension (Table 1 and Fig. 3).

**Table 1:** From 3400 evaluated people, 410 people had abnormal aortic size

Characteristic	Number		
Male	235		
Female	175		
> 60 years old	255		
35–60 years old	139		
< 35 years old	16		
Hypertension	229		
Ascending aorta aneurysm	42	patients > 60 years old	25
		patients with hypertension	23
Coronary artery disease	73		
History open coronary artery surgery	23		
Coronary artery stent	18		
Chronic heart failure	54		
History of stroke	6		
Diabetes mellitus	22		
Bicuspid aortic valve (BAV)	26	patients > 35 years old	16
Aortic valve insufficiency	23		
Aortic valve stenosis	11		
Smokers	22		
Tetralogy of Fallot	1		
Marfan	1		
Aortic dissection	2		
Positive family history of ATAA	5

## Discussion

Among 3400 people studied, 410 (12%) had abnormal aorta size, and 42 (1.2%) were diagnosed with ATAA. Of the 410 people with abnormal aorta size, 56% had high blood pressure, 18% had coronary artery disease, 6% reported a history of open coronary artery surgery, and 4.4% had a history of coronary artery stent. Among 42 patients with ATAA, 60% were over 60 years old, and 55% had hypertension. According to our results, it seems that the most common risk factors of ATAA are advanced age and hypertension. In young people (under 35 years old); however, the most common risk factor was BAV. Out of 16 patients under the age of 35 years old, 12 had BAV, two were pregnant, one had tetralogy of Fallot, and one had aortic dissection.

Overall, aorta size increases with the body size and age, and an elevated aortic size is also associated with male gender [15, 16]. In the study of Comb et al., the range of ascending aortic diameter based on the results of CT scan was reported from 29 to 37.2 mm for females and from 30.8 to 39.1 mm for males [17]. On the other hand, according to echo findings, the normal size of the aorta fluctuates at different ranges [3]. Aneurysm is diagnosed when a part of an artery bulges out of vascular walls usually where the wall is weak. A study emphasized that the size of thoracic aorta aneurysm was larger than 45 mm [17], which was used in this study as a diagnostic parameter for ATAA and for categorizing ascending thoracic aortic diameter. Therefore, in our study, patients with an ascending aorta diameter of greater than 45 mm were considered to have ATAA. These patients were further evaluated for risk factors and potential etiologies.

According to the study of Clure et al., the incidence of TAA has increased from 3.5 to 7.6 per 100,000 persons between 2002 and 2014 [18]. In another study, TAA incidence was reported to be at least 5 to 10 per 100,000 person-years [12]. Linda et al. reported the incidence of TAA as 5.9 per 100,000 person-years [11]. Furthermore, the role of environmental, cultural, and racial factors in the development of aneurysm should be evaluated.

In this study, we investigated the prevalence of ATAA in an Iranian population. Among 3400 Iranians who underwent echo, 410 (12%) showed abnormally elevated aortic size, and 42 (1.2%), based on a diameter of larger than 45 mm, were diagnosed with ATAA, indicating an incidence of 1.2 per 100 persons. The high prevalence of aneurysm in the studied population is clinically significant as an elevated aortic diameter can denote serious problems such as aortic dissection or rupture [18]. As a chronic condition presenting with progressive aortic dilatation due to changes in the extracellular matrix and smooth muscle cells, TAA can be associated with many environmental and genetic causes [19].

The most common type of TAA is degenerative aneurysm which is associated with an advanced age and cardiovascular risk factors such as hypertension and atherosclerosis [19]. Ascending aorta aneurysm often occurs due to cystic medial degeneration which is characterized with elastic fibers degradation and smooth muscle drop out. Medial degeneration weakens the vascular wall and facilitates the formation of aneurysm; a process which is exacerbated by aging and hypertension [20]. Our study confirmed that the most common risk factor of TAA was old age so that 62% of patients with abnormal ascending aorta sizes and 60% of patients with ATAA were over 60 years old. So, age-related degenerative changes could be major causes of aneurysms in our patients; however, this requires further studies.

In our patients, the second risk factor of elevated aortic size was hypertension. In this regard, 56% of patients with abnormal aortic sizes and 55% of those with ATAA had hypertension. The high prevalence of hypertension in the studied population may explain the high incidence of aneurysm. Therefore, it is necessary to implement accurate plans to identify individuals with hypertension and provide them with appropriate treatment and adequate follow up and encourage them to modify their lifestyle.

Cystic medial degeneration, a risk factor for ATAA, is common in patients with connective tissue diseases [20]. Patients with connective tissue syndromes (Marfan, Vascular Ehlers Danlos type IV, Loeys- Dietz, and Familial thoracic aortic aneurysm) and BAV have defects in smooth muscles and the elastic tissue of the aorta, leading to the dilatation of the artery and aneurysm [21]. Marfan syndrome is an autosomal-dominant disorder caused by mutations in the gene encoding fibrillin-1 [22]. More than 100 mutations are involved in the development of Marfan syndrome [23]. In most cases, Marfan-induced aortic aneurysm symmetrically involves the sinuses of Valsalva while ascending aorta and sinotubular junction are relatively preserved [21]. Among the 3400 patients studied here, only one case of Marfan syndrome was detected, who had an ascending aortic size of 46 mm and a history of aortic valve replacement due to severe aortic regurgitation.

The prevalence of BAV in our study was 0.76%. BAV generally affects 1% of the general population and may be associated with ATAA [5]. One of the causes of ATAA in BAV is post-stenotic dilatation [5]. In a study, Nistri et al. evaluated people with normally functioning bicuspid aortic valve and reported that 52% of them had aortic dilatation [24]. Likewise, another study reported an association between BAV and aorta dilatation [25]. Nevertheless, it seems that post-stenotic dilatation is not the only mechanism involved in the aortic dilatation seen in BAV. Cystic medial degeneration can also contribute to the dilatation of the aorta in this condition [26]. So, ATAA can be associated with BAV and may develop late after AVR [27]. In our study, 26 patients (6.3%) with abnormal ascending aortic sizes had BAV, but only six of them showed aortic aneurysm. On the other hand, BAV was the most common cause of ascending aorta enlargement in the patients who had less than 35 years of age. In fact, age-dependent degenerative changes enhance the contributing effects of BAV.

Atherosclerosis is an infrequent cause of ATAA, but it is the predominant etiology of descending thoracic aorta aneurysm [5]. Aortic atherosclerosis can be associated with atherosclerotic changes in coronary arteries. In our study, 18% of patients had a history of coronary artery disease. On the other side, we found that atherosclerosis was a rare cause of aneurysm compared to other risk factors such as hypertension and advanced age.

TAA is usually a silent disease and often is accidentally found during examining for other medical conditions. The symptoms of ATAA are related to progressive AR, local mass effects, and the systemic embolization caused by mural thrombus [28]. Therefore, it is very difficult to estimate the incidence and prevalence of TAA in the general population. Overall, 5.6% of our patients (410) with abnormal aortic sizes had moderate or severe aortic regurgitation. Six of our patients (1.5%) also had a history of stroke. It is worth mentioning that one of the causes of stroke can be systemic embolism due to TAA.

Ascending aorta aneurysm is a very dangerous pathology of the aorta and if not treated in time, can cause potentially fatal complications such as aorta rupture or dissection with the incidence of 10 per 100,000 person-year [29]. The rate of serious complications increases from 10% at 60-mm maximum diameter to 43% at 70-mm [30–33]. Early diagnosis before an increase in the size of aneurysm greatly helps to timely carry out necessary and appropriate treatments to prevent complications.

Among our patients, 56% had hypertension, and 5.4% smoked, indicating the need for lifestyle modifications along with therapeutic interventions. Furthermore, sex is a predictor of aneurysm complications. The control of risk factors and complications is necessary for disease management. The main risk factors of aneurysm include hypertension, COPD, a family history of the disease, advanced age, increased diameter of ascending aorta, male gender, and cigarette smoking [7, 34, 35]. In our study, women constituted 43% of patients; among them two were pregnant. In the present study, average age and the prevalence of hypertension were higher in women than men. Given that the two main risk factors (i.e. advanced age and hypertension) were more pronounced in women, the greater aneurysm growth rate in them can be explained. Therefore, proper planning is needed to improve these patients’ quality of life by encouraging them to adopt a healthy lifestyle.

Patients with dilated aorta should be identified and informed about the risk of aorta dissection and rupture. They also should be educated and guided to adopt appropriate lifestyle modifications. Patients with degenerative ascending TAA should receive a low-fat diet, undergo regular long-term imaging and screening every 6 months. On the other hand, patients with degenerative TAA should meet for imaging evaluations annually when aorta diameter is 40–45 mm and each 6 to 12 months when the diameter is between 45 and 54 mm. In patients with familiar TAA and Marfan syndrome, annual imaging is necessary when the aortic diameter ranges from 35 to 44 mm while biannual evaluation should be performed when the diameter is between 45 and 54 mm [7, 36, 37]. Health planning is essential for regular screening of patients and to estimate health costs by determining the prevalence of ATAA. In addition, it is necessary to determine and manage the main risk factors of the disease. In athletes with an aortic size of greater than 40 mm, low static/low dynamic exercises are recommended [38]. Because blood pressure may increase during physical activity, especially in isometric activities, patients should be cautious about the extent of their activities and avoid strenuous exercises. These patients should also be advised to adopt a healthy lifestyle.

## Conclusion

In this study, we showed that the prevalence of ascending aorta aneurysm in the general population of Iran was about 1.2%. Determining the prevalence and early diagnosis of ATAA is necessary to improve public health and prevent death and costs. In addition, it is necessary to determine and manage the main risk factors of the ATAA. The high prevalence of hypertension may explain the high incidence of aneurysm in our studied population. Therefore, it is necessary to implement an accurate screening plan to identify patients with hypertension and provide appropriate treatment and adequate follow up to patients for prevention of death, increasing health care quality and modify lifestyle of the patients.

## Data Availability

Not applicable.

## Abbreviations

AR: Aortic regurgitation
ATAA: Ascending thoracic aorta aneurysm
AVR: Aortic valve replacement
BAV: Bicuspid aortic valve
COPD: Chronic obstructive pulmonary disease
CTA: Computed Tomography Angiography
CT: Computed Tomography
Echo: Echocardiography
LA: Left Atrium
LV: Left Ventricle
MRA: Magnetic Resonance Angiography
RA: Right Atrium
RV: Right Ventricle
TAA: Thoracic aorta aneurysm
TEE: Trans esophageal echocardiography
TTE: Transthoracic echocardiography
